# Not only reseeder or resprouter plants: Trait syndromes and post‐fire responses of three iconic Mediterranean woody species

**DOI:** 10.1111/plb.70213

**Published:** 2026-04-03

**Authors:** G. Ottaviani, H. Beckett, J. M. Costa‐Saura, E. Agrillo, G. Bonari, C. Calfapietra, E. D'Andrea, P. Fiorucci, M. Lo Cascio, M. Magnani, S. Portarena, C. Sirca, M. Baudena, M. Millan

**Affiliations:** ^1^ Research Institute on Terrestrial Ecosystems (IRET) National Research Council (CNR) Porano Italy; ^2^ National Biodiversity Future Center (NBFC) Palermo Italy; ^3^ School for Climate Studies Stellenbosch University Matieland South Africa; ^4^ Department of Agricultural Sciences University of Sassari Sassari Italy; ^5^ Foundation Euro‐Mediterranean Center on Climate Change (CMCC) Sassari Italy; ^6^ Institute for Environmental Protection and Research (ISPRA) Rome Italy; ^7^ Department of Life Sciences University of Siena Siena Italy; ^8^ CIMA Research Foundation Savona Italy; ^9^ Institute of Geosciences and Earth Resources (IGG) National Research Council (CNR) Turin Italy; ^10^ Institute for Atmospheric Sciences and Climate (ISAC) National Research Council (CNR) Turin Italy; ^11^ Université de Mayotte Dembéni France; ^12^ AMAP, CIRAD, CNRS, INRAE, IRD, Université de Montpellier Montpellier France

**Keywords:** Ecosystem dynamics, fire ecology, functional trait, growth, intraspecific trait variability, ontogeny, resource conservation, species‐specific responses, trait coordination

## Abstract

- Fire can profoundly affect ecosystem dynamics, species distribution and plant traits, especially in open biomes. Post‐fire strategies, namely, resprouters and reseeders, offer a useful framework to examine eco‐evolutionary relationships between plants and fire. However, whether resprouter and reseeder plants are consistently formed by distinct trait coordination (syndromes) and responses to fire at the intraspecific level and when considering the role of ontogeny, remain underexplored. This is a relevant lack as, within‐species, plants can adjust their functioning and trait coordination can vary considerably along ontogeny. To address this gap, we analysed intraspecific trait coordination and post‐fire responses, accounting for the effect of ontogeny in three widely distributed and locally abundant Mediterranean woody species: two resprouters (*Erica arborea*, *Quercus ilex*) and one reseeder (*Cistus salviifolius*).
- We collected 12 plant functional and architectural traits, including intraspecific variability, well related to fire and drought from three sites in Italy. We ran pairwise correlation and multivariate analyses to explore trait syndromes. We conducted linear regressions to examine relationships between fire regime (time since last fire) and trait responses. We then inspected whether fire regime affects key bivariate trait coordination and if ontogeny influences some trait‐fire links.
- Findings are highly species‐specific and generally do not align with *a priori* classification into post‐fire strategies. In most instances, we reveal how either one of the resprouter species exhibits trait patterns more similar to those of the reseeder than to the other resprouter species. Fire can strongly affect trait coordination shaping plant functioning, whereas ontogeny influences a few trait‐fire links for the reseeder species while it has a weak effect on the two resprouter species.
- Our study, while limited to three species and three sites, emphasizes the importance of looking at plant life through a continuous and multidimensional lens which contemplates the inclusion of various sources of within‐species variability. We acknowledge that a category‐based or dichotomous view on plant functional strategies, including post‐fire ones, remains valid and justified when working at coarse scales, whereas it can be much less so for trait‐based analyses at fine scales.

## Graphical abstract

Post‐fire plant functional strategies of Mediterranean woody species can be less fixed than often thought when intraspecific variability of fire‐related traits is considered.

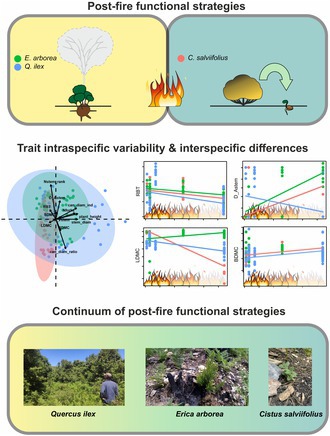

## INTRODUCTION

Fire has occurred on Earth for at least 420 million years (Bowman *et al*. 2009; Pausas & Keeley 2009). As such, fire disturbance represents an eco‐evolutionary force affecting the dynamics of terrestrial ecosystems and biological assemblages from the local up to the regional and global scale (Bond *et al*. 2005; Bond & Keeley 2005; Charles‐Dominique *et al*. 2015; Lamont & He 2017). As primary producers, plants constitute fundamental biotic components of any ecosystem. At the same time, plant organs and tissues (*e.g*., leaf, stem; with distinct traits affecting fire behaviours) – both dead and alive – constitute the fuel necessary for any fire to ignite, burn and spread (Grootemaat *et al*. 2017; Jaureguiberry & Díaz 2023). Consequently, long‐term fire dynamics have shaped the diversity and distribution of plant species, as well as their forms and functions, since the emergence of plant life in terrestrial ecosystems (Bond 2005; Pausas & Keeley 2009, 2023; Keeley *et al*. 2011; Lamont & He 2017).

Fire is particularly relevant in those regions hosting open biomes – such as tropical savannas or mediterranean‐type shrublands (Lamont & He 2017; Pausas *et al*. 2018; Buisson *et al*. 2022). Additionally, many open biomes are associated with rich and often endemic biological diversity (Parr *et al*. 2014; Ottaviani *et al*. 2024). For example, the five globally distributed regions harbouring mediterranean‐type ecosystems are all recognized global biodiversity hotspots (Myers *et al*. 2000), accounting for ~20% of them while occupying less than 5% of the global land surface (Cowling *et al*. 1996). Fire regimes – defined by fire frequency, intensity, extent, seasonality and type (Archibald *et al*. 2013) – are however increasingly altered by human‐induced changes in land use and climate (Klausmeyer & Shaw 2009; Cunningham *et al*. 2024), with potential detrimental impacts on ecosystem functioning and the provision of associated services (Lecina‐Diaz *et al*. 2021). A better understanding of how plant species may respond to these changes is therefore crucial for basic research in plant functional ecology, evolutionary biology and ecosystem functioning.

Plants can exhibit a variety of functional strategies that may facilitate or hinder their capabilities to cope with certain fire regimes (Pausas & Verdú 2005; Paula *et al*. 2016; Lamont & He 2017). Plant species are typically grouped into different plant functional strategies that can inform on plant abilities to deal with biotic and abiotic conditions, thus supposedly promoting or limiting their local persistence and spatial distribution (*e.g*., Raunkiær's life forms; Raunkiær 1934). Among plant strategies tightly linked to post‐fire, the most used ones refer to reseeders and resprouters (Bell *et al*. 1996; Bellingham & Sparrow 2000; Clarke *et al*. 2013; Pausas & Keeley 2014; Pausas *et al*. 2016; Simpson *et al*. 2021). Reseeders are generally killed by fire, yet fire can stimulate their seed release (serotinous plants; Schwilk & Ackerly 2001) and germination following fire (Lamont & He 2017). Consequently, the post‐fire regeneration of these plants is shifted to the next generation. Resprouters can instead cope with fire by protecting vital tissues (*e.g*., meristems, together with storing other resources, such as non‐structural carbohydrates) sheltered by thick barks or below the soil surface that can be eventually used to restore biomass (Clarke *et al*. 2013; Pausas & Keeley 2014; Pausas *et al*. 2016). Hence, post‐fire regeneration involves the persistence of the same individual experiencing the fire event(s). Some plant species can be more flexible than others and can employ both strategies, such as in the case of facultative reseeders or resprouters (Pausas & Keeley 2014; Keeley *et al*. 2024).

Plant species, however, are not homogeneous entities; instead, they are characterized by a variability in their trait values and different trait coordination and combinations that together form syndromes (Reich *et al*. 1999; Agrawal 2020). It has been highlighted how intraspecific trait variability (ITV) can play a crucial role in shaping plant fitness and responses to changes in environmental conditions (Violle *et al*. 2012; Kichenin *et al*. 2013; Jung *et al*. 2014; Siefert *et al*. 2015; Puglielli *et al*. 2024). While ITV is highly species‐ and trait‐specific – that is, some species and traits may be more variable than others – the magnitude of ITV can even exceed that accounted for by interspecific trait differences (Kichenin *et al*. 2013; Liu *et al*. 2023). Additionally, ontogeny can affect trait patterns because different developmental stages can be characterized by distinct trait expression and trait (co)variation (Barthélémy & Caraglio 2007; Dang‐Le *et al*. 2013; Millan *et al*. 2024). Architectural traits, in particular, have been identified as reliable proxies for developmental stages. For instance, the number of forks (Wigley *et al*. 2020; Millan *et al*. 2024) can detect differences in plant strategies along ontogeny, such as allocation to primary and secondary growth, to potentially escape the fire trap between resprouting and non‐resprouting individuals belonging to the same species in savannas (Millan *et al*. 2024). Despite their potential significance, these sources of within‐species trait variation remain largely underexplored in fire ecology and functional ecology of plants – particularly in mediterranean‐type ecosystems.

To address these issues and related gaps, we focus on three woody species widely distributed and locally abundant in the Mediterranean Basin – two resprouters (*Erica arborea*, *Quercus ilex*) and one reseeder (*Cistus salviifolius*) at three sites in Italy. Focusing on plant syndromes and responses, we analyse changes in 12 functional and architectural traits that can estimate plant fitness and development, as well as capturing links between fire regime and plants. We therefore set out to examine whether and to what extent these typical Mediterranean resprouter and reseeder woody species differ in their post‐fire functional strategies in terms of trait coordination and their relationships with fire – considering intraspecific variability and interspecific differences. We ask:

**Q1**. Do resprouter and reseeder species differ in their trait coordination? Within resprouters, are *E. arborea* and *Q. ilex* characterized by similar trait coordination?

**Q2**. Do trait patterns indicate fire‐modulated plant responses to fire regime? Do these patterns differ between resprouter and reseeder species, as well as between the two resprouter species?

**Q3**. Does fire regime affect key bivariate trait coordination relationships?

**Q4**. Do developmental stages – proxied by architectural traits – affect key relationships between functional traits and fire regime?

## MATERIALS AND METHODS

### Study sites

**Fig. 1:**
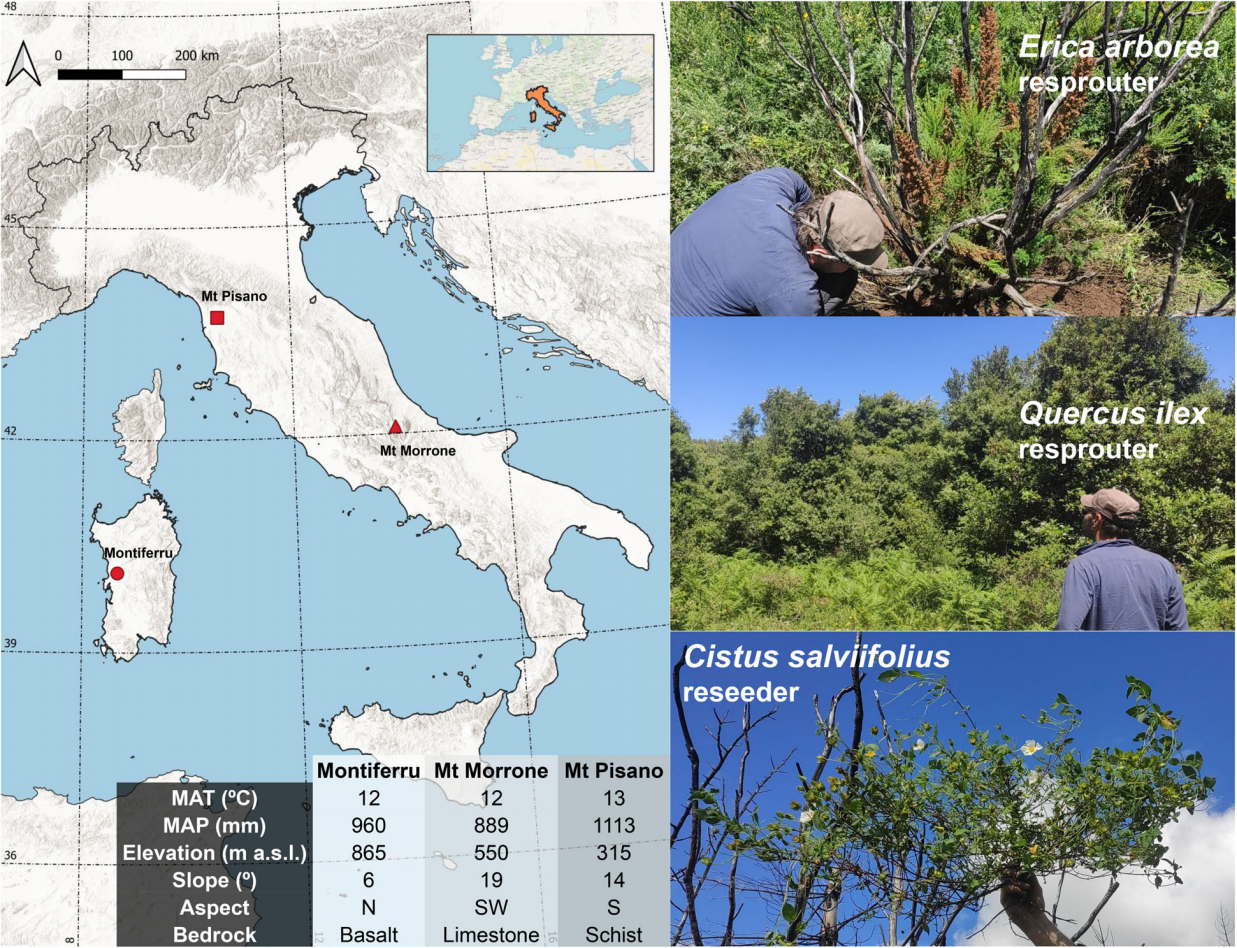
On the left, a map showing the location of the three study sites in Italy, with the main macroclimatic, topographic, and geological characteristics of each site. On the right, the three focal species (photo credit: José Maria Costa‐Saura).

We selected three sites in Italy experiencing mediterranean‐type climate, defined by a marked temperature and precipitation seasonality, with xeric Summers (warm and dry) and mesic Winters (cool and wet). The three sites are: Montiferru (centroid: 40.1662048823 N, 8.6397768366 E), Mt Morrone (centroid: 42.1005229667 N, 13.9231161 E), Mt Pisano (centroid: 43.7288732109 N, 10.5761829773 E) – see Fig. 1. Within each site, we selected sampling areas to collect traits by stratifying for main abiotic variables (*i.e*., elevation, aspect, slope, bedrock type) with the aim to reduce within‐site environmental heterogeneity to better disentangle and estimate the effect of fire regime on trait patterns from other confounding abiotic factors. Edaphically, Montiferru and Mt Pisano sites are on silica‐rich soils, whereas Mt Morrone site is on carbonate‐rich soils (Fig. 1 and Table 1).

**Table 1:** Description of the sampling design for collecting plant traits at the three study sites for the three focal Mediterranean woody species, with fire regime metrics and the number of plant individuals (n) examined in each sampling area.

				fire metrics			resprouter		reseeder
site	soil	sampling area	year burnt	number of fires	time since last fire (yr)	fire return interval (=yrs/(#fires + 1))	*Erica arborea* (n = 40)	*Quercus ilex* (n = 50)	*Cistus salviifolius* (n = 30)
Montiferru	Silica‐rich	Control – holm oak	<1970	0	54	54		10	5
		Control – mixed	<1970	0	54	54		10	
		Two fires	1994, 2021	2	3	18	10	5	10
		Three fires	1983, 1994, 2021	3	3	13.5	5	10	
Mt Morrone	Carbonate‐rich	One fire	2017	1	7	27		10	
		Two fires	2017, 2023	2	1	18		5	
Mt Pisano	Silica‐rich	Control – pine	<1970	0	54	54	10		
		One fire	1998	1	26	27	10		5
		Two fires	1998, 2023	2	1	18	5		10

### Fire regime

We focused on three metrics that were available since the year 1970 for all three study sites, namely the number of fires, fire return interval and time since last fire (Table 1). We could not gather information related to fire intensity and severity because of the paucity of Earth observations' monitoring during the events, given the timespan involved. In addition to this lack of fire severity data, most within‐site sampling areas (differing in fire regime) are adjacent to one another, hindering the use of high‐resolution satellite data.

### Focal species

We selected three focal woody, semi‐deciduous, light‐demanding plant species belonging to two post‐fire strategies and three families (Fig. 1), namely, two resprouters (*Erica arborea* L. – Ericaceae, *Quercus ilex* L. – Fagaceae) and one reseeder (*Cistus salviifolius* L. – Cistaceae) (Tavşanoǧlu & Pausas 2018). *E. arborea* is a large shrub with a conspicuous below‐ground lignotuber or burl storing buds and carbohydrates found both in open and closing habitats (dying off in closed canopies) like maquis, which is capable of vigorous resprouting but can also emerge from seeds after fire (Tavşanoǧlu & Pausas 2018). *Q. ilex* is a long‐lived tree or large shrub with high resprouting abilities that can present tuberized below‐ground structures (mainly lignotuber) thriving in open habitats like garrigue, and without disturbance, can close the habitat canopy. *C. salviifolius* is a relatively short‐lived and small shrub representative of open shrublands – for example, it gives the name to a specific vegetation type in southern France called ‘cistaie’ (Rameau *et al*. 2008). These species are representative of some iconic Mediterranean vegetation types and are often locally abundant and with a broad geographic range in the Basin.

### Plant trait sampling

Fieldwork activity occurred during late Spring and early Summer 2024, corresponding to the vegetative and flowering peak in the study sites. Within each site, we selected one sampling area for each fire regime condition. We could not include within‐site replicates for fire regime conditions – this constraint was primarily due to maintaining other within‐site environmental variables as similar as possible while having variation in fire regime. We replicated the same sampling design across the three sites, adjusting our sampling to local conditions (Table 1). Each within‐site sampling area is constituted by a circular plot of ~300 m^2^ (~10 m radius) wherein we sampled 5–10 individuals for each focal species – depending on species occurrence and abundance.

Overall, we sampled 120 individual plants (40 *E. arborea*, 50 *Q. ilex*, 30 *C. salviifolius*) for collecting trait data in the field and in the lab (Table 1). The selected 12 functional and architectural traits are related to the three key components of plant fitness, namely, informing on growth, survival and reproduction (Violle *et al*. 2007) – see Table 2. These traits can capture specific plant functions as well as how plants may respond to variations in environmental conditions (Weiher *et al*. 1999; Violle *et al*. 2007) – in this study focused on mediterranean‐type ecosystems, we were mainly interested in fire and drought. We followed standard protocols and specialized literature to select and collect traits – see references reported in Table 2. For those traits for which standard protocols were not available, we devised our own sampling and scoring protocol; these traits are generally count or proportion variables estimated in the field or calculated from other (trait) values (can_diam_ratio, D_Astem, G_Vstem). Regarding the two architectural traits, informing on individual developmental stage, we recorded (i) number of stems (Nstem_rank), especially relevant for the reseeder shrub *C. salviifolius*, and (ii) number of successive forks (Nforks), particularly relevant for the resprouter species *E. arborea* and *Q. ilex* (Table 2).

**Table 2:** The 12 functional and architectural traits included in this study, ordered by their links to plant fitness (growth, survival, reproduction), with units and abbreviations as used in the figures, sampled organ(s), what to collect and how to score the trait, main functions captured by the trait (informing on fire‐ and drought‐related plant strategies) with key references (handbooks of standard protocols for collecting traits are in italics).

trait name (units)	abbreviation	plant organ	what to collect and how to measure	link to plant fitness	main plant functions	key references
Basal stem diameter (cm)	stem_diam	Stem	Diameter of the main stem at its base measured using a calliper or a metre tape	Growth	Above‐ground biomass allocation; structural support	Moncrieff *et al*. (2011); Millan *et al*. (2024)
Canopy diameter of the entire individual (cm)	can_diam_ind	Stem; leaf	Diameter (average of the two main dimensions) measured using a metre tape	Growth	Light capture; Above‐ground biomass allocation; productivity	Prescott (2002); Jucker *et al*. (2015)
Relative bark thickness (dimensionless)	RBT	Stem (base)	Ratio between bark thickness (of the main stem measured at its base) and bole diameter, multiplied ×100	Survival	Protection from disturbances; resource conservation	Midgley & Lawes (2016); Pellegrini *et al*. (2023); *Wigley et al. (* 2020 *)*
Proportion between dead and alive stems (%)	D_Astem	Stem	Estimated percentage between dead and alive stems	Survival	Disturbance damage; mortality/vitality	This study
Below‐ground coarse organ dry matter content (mg g^−1^)	BDMC	Thick root; lignotuber	Ratio between the oven‐dried mass (at 60°C for 72 h) and the fresh mass, weighted with a balance, of the below‐ground coarse organ (lignotuber, thick root) sampled with a trephor a few centimetres below the soil surface	Growth; Survival	Resource (water) conservation; plant lifespan; C‐allocation; structural support	Ottaviani *et al*. (2022); Midolo *et al*. (2024)
Leaf dry matter content (mg g^−1^)	LDMC	Leaf	Ratio between the oven‐dried mass (at 60°C for 72 h) and the fresh mass, weighted with a balance, of 5–10 undamaged leaves per sampled individual collected along the main stem	Growth; Survival	Resource (water) conservation; leaf and plant lifespan; flammability; Resistance to herbivory; C‐allocation	*Cornelissen et al. (* 2003 *)*; Kazakou *et al*. (2006); Alam *et al*. (2025)
Stem dry matter content (mg g^−1^)	SDMC	Stem (base)	Ratio between the oven‐dried mass (at 60°C for 72 h) and the fresh mass, weighted with a balance, of the main stem sampled at its base	Growth; Survival	Resource (water) conservation; plant lifespan; flammability; C‐allocation; structural support	Adapted from *Cornelissen et al. (* 2003 *)*
Ratio between canopy diameter of the main stem and that of the entire individual (dimensionless)	can_diam_ratio	Stem; leaf	Ratio between the measured diameter of the main stem canopy and that of the whole individual	Growth; Survival	Above‐ground biomass allocation; Light capture; response to disturbance	This study
Proportion between generative and vegetative stems (%)	G_Vstem	Stem bearing flowers and/or fruits	Assessed percentage between stems bearing sexual reproduction organs (flowers, fruits) and those without any sexual reproduction organs	Reproduction	Main reproduction type; generative vs vegetative reproduction effort	This study
Plant height (cm)	plant_height	Stem	Plant height measured using a metre tape as the vertical distance between the soil surface and the highest living plant organ (*e.g*., leaf, flower)	Growth; Reproduction	Above‐ground biomass allocation and vertical space occupancy; light capture; escape from disturbance trap(s); dispersal distance potential	*Cornelissen et al. (* 2003 *)*; Moles *et al*. (2009); Thomson *et al*. (2011); Wakeling *et al*. (2011)
*Architectural traits to estimate ontogeny*						
Number of stems (#)	Nstem_rank	Stem	Number of stems emerging from the ground (*e.g*., from the lignotuber), transformed as rank variable: 1 = 1 stem, 2 = 2–3 stems, 3 = 4–5 stems, 4 = 6–10 stems, 5 = 10+ stems	Growth; Survival; Reproduction	Proxy for developmental stage in shrubs; above‐ground biomass allocation; response to disturbance	*Wigley et al. (* 2020 *)*
Number of successive forks (#)	Nforks	Stem	Number of successive forks along the main stem	Growth; Survival; Reproduction	Proxy for developmental stage in trees; above‐ground biomass allocation; escape from disturbance trap(s); Light capture	Barthélémy & Caraglio (2007); Wigley *et al*. (2020); Millan *et al*. (2024)

### Data analysis

We performed all statistical analyses in R version 4.4.2 (2024). For **Q1**, we conducted a multivariate analysis (Principal Component Analyses [PCA]) on the 12 traits, plotting the occupancy of each species (as well as the occupancy of the three sites in the functional space, which showed high overlapping; see Fig. S2) – using functions available in the *factoextra* package (Kassambara & Mundt 2020). We tested whether the three focal species differed in the multivariate functional space by running a PERMANOVA test (with 999 permutations), using the *vegan* package (Oksanen *et al*. 2024). Then, to examine pairwise trait correlations, we ran correlograms using the *corrplot* package (Wei & Simko 2024). Before running the models devised to address **Q2–Q4**, we checked for collinearity among the three fire regime metrics, finding them to be strongly correlated in our dataset (Fig. S3). As we are interested in post‐fire plant trait syndromes and responses, we chose time since last fire as the predictor in the following models.

For **Q2**, to study single‐trait responses to variation in fire regime, we ran linear regressions setting the fire regime metric as the predictor and a functional trait as the response variable, controlling for species identity effect (*i.e*., set as grouping factor). Based on previous correlation analysis and PCA results, we focused on those traits tending to form independent functional dimensions, ending up with seven trait‐fire relationships.

For **Q3**, to inspect whether changes in fire regime affect bivariate trait coordination, we selected key relationships based on previous analyses as well as on their link to plant fitness and trait association with fire and drought (Table 2) – working with each species separately. Using linear regressions, we examined the following four key fitness‐related trait relationships: basal stem diameter *versus* plant height (informing on growth), relative bark thickness *versus* proportion between dead and alive stems (informing on survival), plant height *versus* proportion between generative and vegetative stems (informing on reproduction), and stem dry matter content *versus* ratio between canopy diameter of the main stem and that of the entire individual (informing on structural support and C‐allocation strategies), setting time since last fire as the interaction term.

For **Q4**, to investigate whether ontogeny affects trait‐fire links, we selected four key relationships based on previous analyses as well as on their tight connection to plant fitness and trait association with fire and drought (Table 2) – working with each species separately. In linear regressions, we set time since last fire as the predictor and a functional trait as the response variable (plant height, relative bark thickness, proportion between generative and vegetative stems, leaf dry matter content), controlling for the effect of developmental stages proxied by architectural traits (*i.e*., set as interaction term). For analyses devised to address **Q2–Q4**, we applied ordinary least squares regressions, employing functions available in *stats* (R 2024), *smatr* (Warton *et al*. 2012) packages. Model assumptions of linearity, normality and homoscedasticity of residuals were accommodated after log10‐transformation of trait values (see Fig. S1 for raw trait data distribution of the three focal species).

## RESULTS

**Fig. 2:**
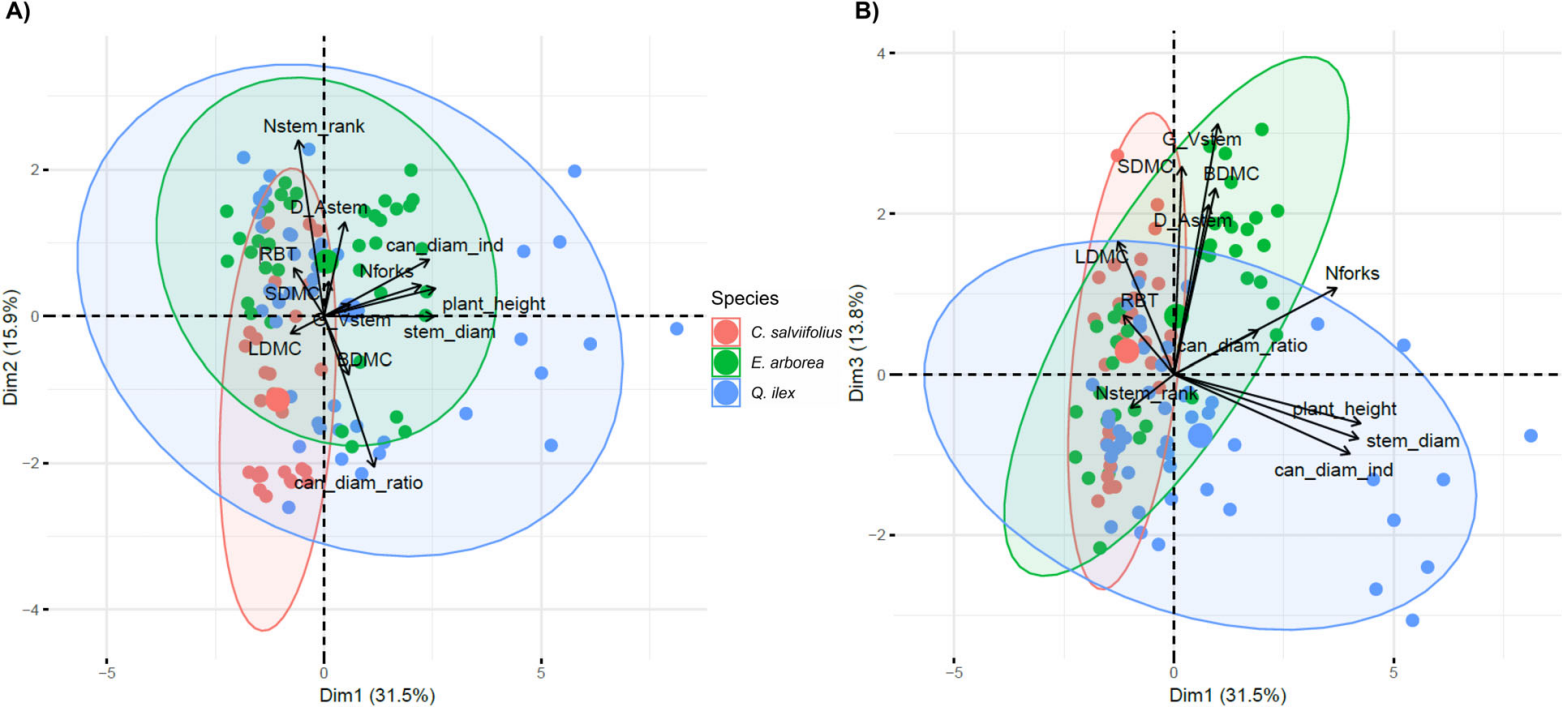
Results from Principal Component Analysis (PCA) conducted on the 12 functional and architectural traits, with the occupancy of the three focal Mediterranean woody species in the multivariate space (95% CIs ellipses). Biplots are related to PCA 1–2 (A) and PCA 1–3 (B) dimensions, with species' centroids in the multivariate space identified by larger points.

**Fig. 3:**
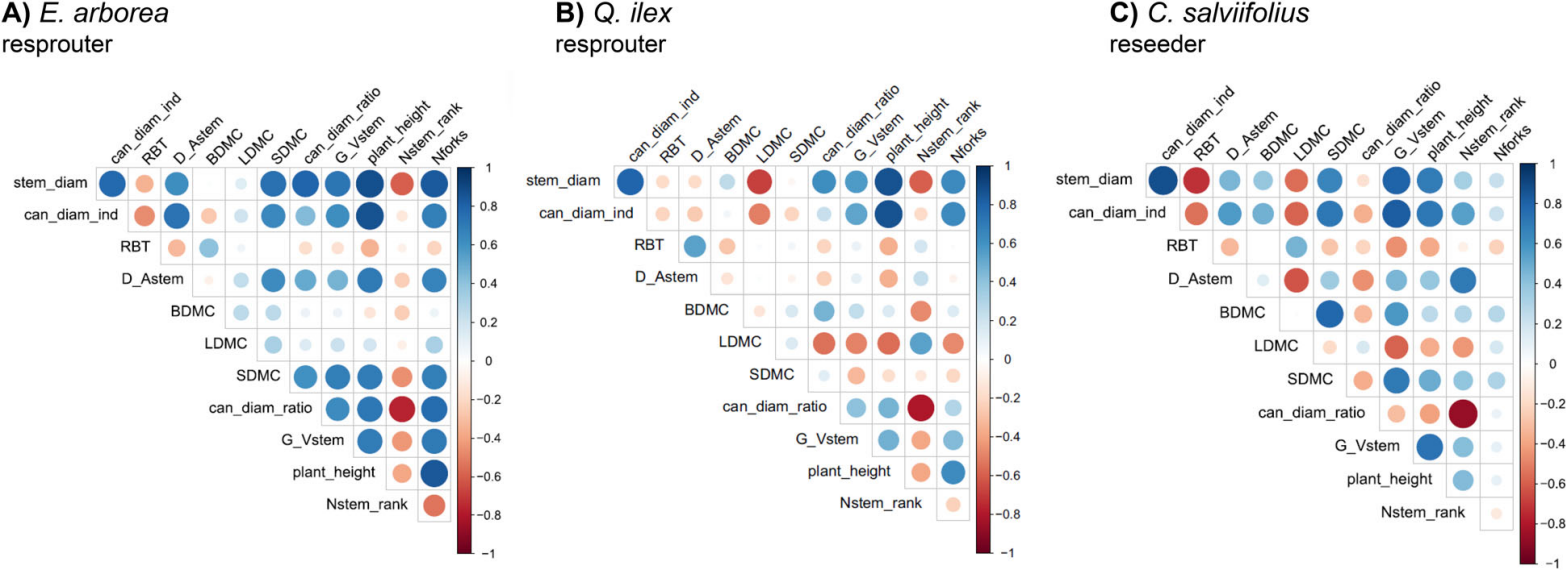
Correlation matrices (based on Spearman rho) between the 12 plant functional and architectural traits for the three focal Mediterranean woody species: *Erica arborea* (A), *Quercus ilex* (B), *Cistus salviifolius* (C).

Concerning **Q1**, the three focal woody species exhibit distinct multivariate and bivariate trait coordination (Figs 2 and 3). The first PCA dimension (variance explained = 31.5%) is primarily related to growth and developmental traits, whereas the second PCA dimension (variance explained = 15.9%) is mainly associated with allocation to single vs multiple stems and related canopy occupancy for light capture. The third PCA dimension (variance explained = 13.8%) is instead mostly shaped by the dry matter content of different plant organs, allocation to sexual reproduction, and stem mortality.

The three species show a high degree of overlapping in the multivariate functional space identified by the 12 traits, especially along the first two dimensions (Fig. 2A), yet individuals of *E. arborea* and *C. salviifolius* are characterized by a high dry matter content of different plant organs as well as a high proportion of dead stems and of stems bearing sexual reproduction structures (observable when PCA1‐3 are plotted; Fig. 2B and Fig. S2). *E. arborea* is also characterized by a tight trait syndrome (as is *C*. *salviifolius*), whereas *Q. ilex* is less coordinated – as further highlighted by the pairwise trait co‐variation (Fig. 3). Growth and architectural traits show similar patterns in the two resprouter species, while differing from those revealed for the *C. salviifolius*. However, other resource‐conservation trait coordination indicates that the resprouter *E. arborea* behaves more similarly to the reseeder *C. salviifolius* than to the other resprouter *Q. ilex* – as in the case of SDMC, which is very strongly integrated and tightly linked to most traits in *E. arborea*, and to a lesser degree, in *C. salviifolius*, while being almost completely decoupled in *Q. ilex*. Conversely, LDMC is highly coordinated in both *C. salviifolius* and *Q. ilex*, whereas it tends to form an independent axis in *E. arborea*. The PERMANOVA test confirmed that the three species differ in their multivariate functional space occupancy, hence syndromes (Full model: *F*‐value = 14.41, R^2^ = 0.20***; *C. salviifolius* vs *E. arborea*: *F*‐value = 10.38, R^2^ = 0.14***; *C. salviifolius* vs *Q. ilex*: *F*‐value = 21.27, R^2^ = 0.22***; *E. arborea* vs *Q. ilex*: *F*‐value = 10.09, R^2^ = 0.11***).

**Fig. 4:**
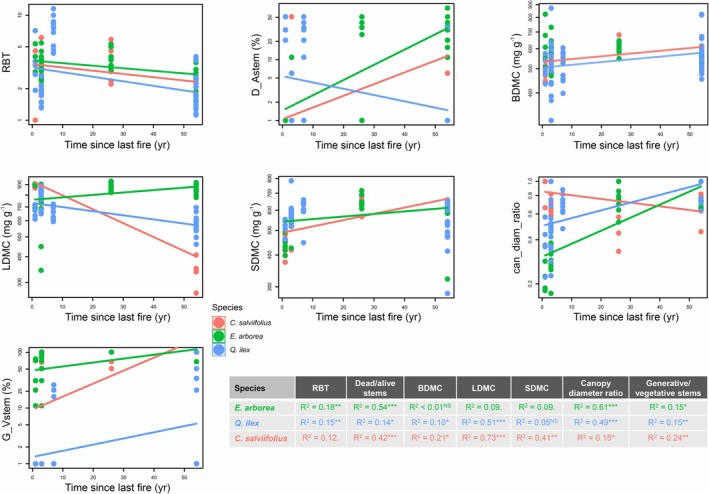
Intraspecific responses of the selected plant functional traits to time since last fire for the three focal Mediterranean woody species. Regression lines are reported for significant and marginally significant relationships – refer to embedded table for models' R^2^ and *P*‐values (*** ≤ 0.001; ** ≤ 0.01; * ≤ 0.05; ≤0.1; NS = not significant > 0.1). See Table 2 for trait abbreviations and Data S1 for models' summary statistics.

Regarding **Q2**, across the seven relationships between time since last fire and functional traits selected in this analysis, the intraspecific patterns are generally strong, indicating fire‐modulated plant trait responses (Fig. 4). Out of the examined 21 relationships, 16 of these are significant or highly significant (with 3 marginally significant relationships), with high to very high variability explained in the models (R^2^ values spanning from 0.10 up to 0.73; Fig. 4). More than half of the trait‐fire relationships indicate that reseeder and resprouter plants may exhibit more similar responses to fire between them than the two resprouter species do. For example, LDMC values of *C. salviifolius* and *Q. ilex* decline with time since last fire, while *E. arborea* exhibits the opposite pattern (marginally significant). D_Astem instead increases as time passes since the last fire in *E. arborea* and *C. salviifolius*, while the opposite happens for *Q. ilex*.

In relation to **Q3**, fire regime plays an interacting role in shaping a few key bivariate functional trait relationships (Table 3). Specifically, time since last fire has a negative interaction effect on the relationship between pairs of (i) growth traits for *E. arborea* and *C. salviifolius*, (ii) survival traits for *Q. ilex*, and (iii) support and C‐allocation traits for *E. arborea* and *Q. ilex*. For the positive relationship between plant height and allocation to sexual reproduction, time since last fire has an interacting positive effect in *Q. ilex* (marginally) and a negative one on *C. salviifolius*. Additionally, time since last fire has a weakly positive interacting effect on the negative relationship between survival traits in *E. arborea*.

**Table 3:** Intraspecific changes in key bivariate trait coordination shaping plant fitness considering the interaction with time since the last fire for the three focal Mediterranean woody species (resprouter: *Erica arborea*, *Quercus ilex*; reseeder: *Cistus salviifolius*).

pairwise trait relationship	species	interaction term: *time since last fire*		functional link to plant fitness
		estimate (SE)	adjusted R^2^, significance	
plant_height~stem_diam	*Erica arborea*	**−0.008 (0.003)**	0.90**	Growth
	*Quercus ilex*	−0.0002 (0.002)	0.84	
	*Cistus salviifolius*	**−0.011 (0.005)**	0.53*	
D_Astem~RBT	*Erica arborea*	***0.038 (0.020)***	***0.55***	Survival
	*Quercus ilex*	**−0.029 (0.007)**	0.50***	
	*Cistus salviifolius*	−0.005 (0.032)	0.36	
G_Vstem~plant_height	*Erica arborea*	−0.015 (0.015)	0.35	Reproduction
	*Quercus ilex*	***0.025 (0.012)***	***0.28***	
	*Cistus salviifolius*	**−0.092 (0.033)**	0.64**	
can_diam_ratio~SDMC	*Erica arborea*	**−0.010 (0.004)**	0.65*	Support, C‐allocation
	*Quercus ilex*	**−0.011 (0.004)**	0.61*	
	*Cistus salviifolius*	0.006 (0.021)	0.13	

The effect of the interaction term on the four selected bivariate trait relationships is reported as estimate, its standard error (SE), adjusted R^2^ of the full model and significance. Bold text in the model summary statistics identifies a significant effect (*P*‐values: *** ≤ 0.001; ** ≤ 0.01; * ≤ 0.05) and bold italics recognizes a marginally significant effect (*P*‐value: ≤0.1) of the interaction term. See Table 2 for trait abbreviations and Data S1 for models' summary statistics.

As for **Q4**, ontogeny – proxied by architectural traits – affects few key trait‐fire relationships, mainly in *C. salviifolius* (reseeder) and, to a lesser degree, in *E. arborea* (resprouter), while it has no detectable effect on *Q. ilex* plants (Table 4). Developmental stage has a negative interacting effect on the positive relationship between time since last fire and primary growth for *E. arborea*, and allocation to sexual reproduction for *C. salviifolius*. Developmental stage also has a negative interaction effect on the negative relationship between time since last fire and LDMC for *C. salviifolius*.

**Table 4:** Interaction effect of ontogeny (*i.e.*, developmental stage proxied by number of successive forks in the resprouters *Erica arborea* and *Quercus ilex* and by number of stems in the reseeder *Cistus salviifolius*) on selected relationships between traits tightly linked to plant fitness and fire regime (time since last fire).

trait‐fire regime relationship	species	interaction term: *ontogeny*		functional link to plant fitness
		estimate (SE)	adjusted R^2^, significance	
plant_height ~ time since last fire	*Erica arborea*	**−0.002 (0.001)**	0.72*	Growth
	*Quercus ilex*	0.0005 (0.001)	0.60	
	*Cistus salviifolius*	−0.002 (0.001)	0.22	
RBT ~ time since last fire	*Erica arborea*	0.0003 (0.001)	0.24	Survival
	*Quercus ilex*	0.0005 (0.002)	0.15	
	*Cistus salviifolius*	−0.002 (0.001)	0.10	
G_Vstem ~ time since last fire	*Erica arborea*	−0.002 (0.002)	0.15	Reproduction
	*Quercus ilex*	0.004 (0.003)	0.27	
	*Cistus salviifolius*	**−0.02 (0.006)**	0.38*	
LDMC ~ time since last fire	*Erica arborea*	−0.0001 (0.0005)	0.06	Water conservation, Flammability
	*Quercus ilex*	<−0.0001 (<0.0001)	0.49	
	*Cistus salviifolius*	**<−0.001 (<0.0001)**	0.83**	

The effect of the interaction term is reported as estimate, its standard error (SE), adjusted R^2^ of the full model and significance. Bold text in the model summary statistics identifies a significant effect of the interaction term (*P*‐values: ** ≤ 0.01; * ≤ 0.05). See Table 2 for trait abbreviations and Data S1 for models' summary statistics.

## DISCUSSION

### Species‐specific trait syndromes of the focal Mediterranean woody plants: not only resprouter or reseeder post‐fire strategies

The concept and classification into plant functional strategies – such as post‐fire ones examined here – have contributed to refining the understanding of plant life functioning and distribution at different spatio‐temporal scales (Raunkiær 1934; Grime 1977; Bellingham & Sparrow 2000; Simpson *et al*. 2021). However, while this broad *a priori* classification comes with many benefits when generalizing into main and easy‐to‐identify categories, which is particularly desirable when working at coarser scales, it may also have shortcomings at finer scales. Here, by inspecting how within‐species trait coordination shapes functional syndromes, we identify how individual plants belonging to different species but to the same post‐fire strategy (resprouter) do not necessarily have more similar functional trait syndromes than individuals belonging to another strategy (reseeder) do. Some plant species can be more flexible than others and employ both strategies, as seen in facultative reseeders or resprouters (Pausas & Keeley 2014; Keeley *et al*. 2024).

Our analysis indicates that the three focal Mediterranean woody species tend to overlap their occupancy in the multivariate functional space. However, the resprouter *E. arborea* and the reseeder *C. salviifolius* are characterized by consistently denser below‐ground coarse organs, stems, and leaves, as well as by a higher proportion of dead and sexually reproducing stems than the other resprouter *Q. ilex* does (Figs 2 and 3). Additionally, because *Q. ilex* has a much longer lifespan, likely in conjunction with a slower ontogeny, than the other two species, our sampling may have captured less developed individuals in this species. Overall, our trait coordination results imply that there is more than one way to be a resprouter (or a reseeder) (Clarke *et al*. 2013; Wigley *et al*. 2020); *E. arborea* may, for specific functions, ‘behave’ more as the reseeder *C. salviifolius* than as the other resprouter species.

### Fire plays a key role in shaping trait responses and coordination of plant functions for the focal Mediterranean woody species

The three species show that variation in fire regime strongly affects several plant functional traits related to fire and drought (Fig. 4). Some of the observed patterns likely indicate fire‐adaptive strategies, such as relatively thinner bark, higher allocation to sexual reproduction and less sclerophyllous leaves (in *Q. ilex* and *C. salviifolius*) as time passes since the last fire event. Results about RBT suggest that soon after a fire, plants – young individuals in *C. salviifolius*, new resprouts in *E. arborea* and *Q. ilex –* tend to allocate disproportionately more to bark for a given size and may potentially be better protected from another disturbance event (Pausas 2015). Regarding lower allocation to sexual reproduction soon after a fire, this result may indicate that sampled individuals were too young and undeveloped to successfully produce flowers and fruits. At the same time, under these circumstances, plants tend to have more flammable leaves, which may generate a positive feedback loop for fire occurrence (Pellegrini *et al*. 2023; Alam *et al*. 2025; Dai *et al*. 2025). Noteworthily, the weakly positive increasing pattern of LDMC with time since last fire revealed for *E. arborea* may also be linked to the different leaf anatomy and morphology of this species (needle‐like) compared with the broad‐leaved *Q. ilex* and *C. salviifolius* that may therefore have different ecophysiological means to cope with hydraulic balance and fire. Still, we acknowledge that our findings can also be associated with other unaccounted for drivers, such as drought or herbivory (*e.g*., Iozia *et al*. 2023). We suggest drought to be especially relevant here as it tends to correlate negatively with time since last fire, since the ecosystem is more open (thus more sun‐exposed and drier) after a fire. This may have largely contributed to plants having more water‐conservative strategies, for example, relatively thicker barks and denser leaves (Richardson *et al*. 2015; Ottaviani & Marcantonio 2021).

Also in the analysis of trait‐fire relationships, we revealed how plant trait responses tend not to align with the resprouter and reseeder *a priori* classification. This finding further corroborates the idea that these post‐fire strategies can be more nuanced and flexible when considering intraspecific variability. We found that for most of the studied relationships, individuals of resprouter species, either *E. arborea* or *Q. ilex*, exhibit trait responses more similar to those emerging for the reseeder *C. salviifolius* than to the other resprouter – as in the case of proportion of dead over alive stems, BDMC, LDMC, SDMC and proportion of generative over vegetative stems (Fig. 4).

Such conflation between resprouter and reseeder strategies also appears in the bivariate trait coordination related to the selected main plant functions (growth, survival, reproduction, support and C‐allocation; Table 3). Of the four trait relationships, patterns are highly species‐specific and do not follow the post‐fire strategy classification. For instance, time since last fire has a negative interaction effect on the allometric relationship between basal stem diameter and plant height (Moncrieff *et al*. 2011; Millan *et al*. 2024) in the resprouter *E. arborea* and reseeder *C. salviifolius*, implying that, as time passes since the last fire, height gain through primary growth declines in favour of secondary growth. However, this effect is not detectable for *Q. ilex* – this lack of pattern may also be attributable to the longevity of this species. Even when both resprouter species are affected by the interaction with time since last fire, the basic bivariate relationship either has an opposite direction, or the interaction has an opposite effect – further indicating highly species‐specific functional adjustments unrelated to the post‐fire strategy.

### Ontogeny affects trait‐fire relationships for reseeder plants, but not those of resprouters in the focal Mediterranean woody species

Developmental stage, proxied by architectural traits (Barthélémy & Caraglio 2007; Millan *et al*. 2024), plays almost no role in shaping trait‐fire relationships for the two resprouter species. In these plants, only for the positive link between time since last fire and plant height in *E. arborea*, ontogeny has a strongly negative interaction effect – implying that as time passes since the last fire, individuals can advance through their ontogeny, reducing their height gain as they mature. This finding may also suggest that after an initial spike in primary growth to overcome a potential fire trap (as observed in tropical savannas; Wakeling *et al*. 2011), the individuals of *E. arborea* may relax their height gain. Still, flame height differs considerably between mediterranean‐type shrublands and tropical savannas, hence this inference would require further scrutiny. For *Q. ilex*, the lack of interaction effect of ontogeny on the selected trait‐fire relationships further supports the hint that, for this long‐lived and slow‐developing species, our sampling captured only a few mature individuals. The lack of developmental variability in our sampling for this long‐lived species calls for future studies to capture a larger ontogenetic spectrum. For the reseeder *C. salviifolius*, ontogeny instead affects relationships between fire and plant sexual reproduction, water conservation, flammability. On the one hand, the negative interaction effect of ontogeny on the positive allocation to sexual reproduction as time passes since the last fire may be related to a few senescing individuals (with a reduced or null ability to reproduce sexually) of this relatively short‐lived and fast‐developing species. On the other hand, the negative interaction effect of ontogeny on the negative relationship between LDMC and time since last fire may be associated with the closing of the habitat canopy. Thus, the more developmentally advanced individuals of this species are considerably less conservative in their water use efficiency and less flammable as time passes since the last fire (Alam *et al*. 2025; Dai *et al*. 2025).

### Concluding remarks: limitations and potential implications

Our findings, while confined to three species and three sites in Italy, stress the need to look at plant life through a continuous multidimensional lens (Carta *et al*. 2025). Results observed in our focal species align with the framework proposing a biological and functional continuum between resprouters and reseeders shaping plant persistence (Pausas & Keeley 2014; Pausas *et al*. 2016). We call for a more widespread use of this approach, also when dealing with plant functional strategies other than post‐fire, especially in local fine‐scale studies (*e.g*., Ottaviani *et al*. 2025). At the same time, we acknowledge the usefulness of discrete classification of broadly defined plant functional strategies in coarse‐scale analyses for which gaining such detailed information of plant life forms is currently out of reach.

Our study may be relevant for applied purposes, such as management (including conservation, restoration, risk mitigation), and may offer insights relevant to predictions of short‐ to long‐term ecosystem dynamics. Providing information gathered at local scales on how plants may functionally cope and respond to variation in fire regimes, including different sources of ITV (phenotypic plasticity; Siefert *et al*. 2015, ontogeny; Millan *et al*. 2024), can assist in refining predictions of vegetation dynamics – especially when focusing on locally abundant and geographically widely distributed species. For example, modelling works found that emergent relationships between plant traits, especially those connected to post‐fire responses, can lead to the existence of alternative states, including in mediterranean‐type ecosystems (Magnani *et al*. 2023). Furthermore, the interaction with climate change and fires acting on such responses can lead to shifts between ecosystem states in the Mediterranean Basin (Baudena *et al*. 2020), possibly leading towards more flammable open ecosystems.

## AUTHOR CONTRIBUTIONS

GO conceived the original research idea, devised the conceptual and analytical framework, planned the sampling design, collected the trait data in the field and the lab, conducted the statistical analysis, wrote the first draft of the manuscript and led its revision process. HB, MB, MMi contributed to the development and refinement of the research idea with inputs on the conceptual and analytical framework as well as on the drafting of the manuscript; MMi contributed also to the rationale behind trait selection and scoring, and to figure and table making. JMCS assisted with field‐ and lab‐activities to collect trait data and with graphics production. ED, MLC, MMa, SP, CS assisted with field‐ and lab‐activities to collect trait data. All authors contributed to revisions of the manuscript.

## CONFLICT OF INTEREST

The authors have no conflict of interest to declare. No AI tools were used.

## Supporting information


**Fig. S1.** Distribution of the 12 functional and architectural traits for the three focal Mediterranean woody species: *Cistus salviifolius* (reseeder), *Erica arborea* (resprouter), *Quercus ilex* (resprouter).
**Fig. S2.** Principal component analysis (PCA; upper biplot refers to dimensions 1–2, lower biplot to dimensions 1–3) ran on the 12 functional and architectural traits showing the occupancy (convex hull) of the three study sites in the multivariate space. On the right panel, trait loadings (correlation) on the five main dimensions of the PCA. MF, Montiferru; MM, Mt Morrone; MP, Mt Pisano.
**Fig. S3.** Correlation (Sperman's rho) between the three fire metrics used in this study: time since last fire event (last_fire), number of fires (Nfires), fire return interval (FRI).
**Data S1**. Summary Statistics Of Models Devised To Address Research Questions 2–4.

## Data Availability

The dataset used in this research is publicly available from the Figshare Digital Repository: https://doi.org/10.6084/m9.figshare.31150144 (Ottaviani *et al*. 2026).
