# Supplementary material for: Not only reseeder or resprouter plants: Trait syndromes and post‐fire responses of three iconic Mediterranean woody species
Source: Plant Biol (Stuttg). 2026 Apr 3;28(5):1399–410. doi: 10.1111/plb.70213 (PMC13358687; doi:10.1111/plb.70213)
Supplement: Supplementary file 1 — Fig. S1. Distribution of the 12 functional and architectural traits for the three focal Mediterranean woody species: Cistus salviifolius (reseeder), Erica arborea (resprouter), Quercus ilex (resprouter). Fig. S2. Principal component analysis (PCA; upper biplot refers to dimensions 1–2, lower biplot to dimensions 1–3) ran on the 12 functional and architectural traits showing the occupancy (convex hull) of the three study sites in the multivariate space. On the right panel, trait loadings (correlation) on the five main dimensions of the PCA. MF, Montiferru; MM, Mt Morrone; MP, Mt Pisano. Fig. S3. Correlation (Sperman's rho) between the three fire metrics used in this study: time since last fire event (last_fire), number of fires (Nfires), fire return interval (FRI). Data S1. Summary Statistics Of Models Devised To Address Research Questions 2–4. [file PLB-28-1399-s001.docx]

**SUPPLEMENTARY MATERIAL FOR**

***Not only reseeder or resprouter plants: trait syndromes and post-fire responses of three iconic Mediterranean woody species***

Gianluigi Ottaviani^1,2^*, Heath Beckett^3^, José Maria Costa-Saura^2,4,5^, Emiliano Agrillo^6^, Gianmaria Bonari^2,7^, Carlo Calfapietra^1,2^, Ettore D’Andrea^1,2^, Paolo Fiorucci^2,8^, Mauro Lo Cascio^4,5^, Marta Magnani^2,9^, Silvia Portarena^1,2^, Costantino Sirca^2,4,5^, Mara Baudena^2,10#^, Mathieu Millan^11,12#^

^1^ Research Institute on Terrestrial Ecosystems (IRET), National Research Council (CNR), Porano, Italy

^2^ National Biodiversity Future Center (NBFC), Palermo, Italy

^3^ School for Climate Studies, Stellenbosch University, Matieland, South Africa

^4^ Department of Agricultural Sciences, University of Sassari, Sassari, Italy

^5^ Foundation Euro‐Mediterranean Center on Climate Change (CMCC), Sassari, Italy

^6^ Institute for Environmental Protection and Research (ISPRA), Roma, Italy

^7^ Department of Life Sciences, University of Siena, Siena, Italy

^8^ CIMA Research Foundation, Savona, Italy

^9^ Institute of Geosciences and Earth Resources (IGG), National Research Council (CNR), Turin, Italy

^10^ Institute for Atmospheric Sciences and Climate (ISAC), National Research Council (CNR), Turin, Italy

^11^ Université de Mayotte, Dembéni, France

^12^ AMAP, Université de Montpellier, CIRAD, CNRS, INRAE, IRD, Montpellier, France

* corresponding author: Gianluigi Ottaviani (gianluigi.ottaviani@gmail.com)

^#^ senior authors

**SUPPLEMENTARY MATERIAL 1**

DATA DESCRIPTION

**
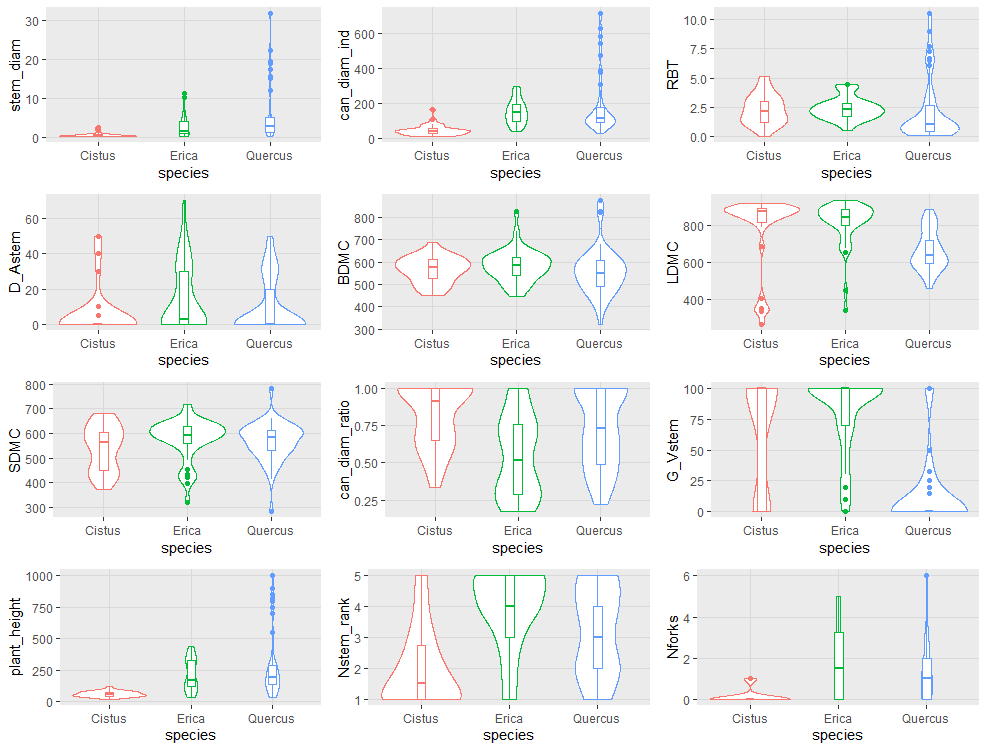
**

**Figure I.** Distribution of the 12 functional and architectural traits for the three focal Mediterranean woody species: *Cistus salviifolius* (reseeder), *Erica arborea* (resprouter), *Quercus ilex* (resprouter).


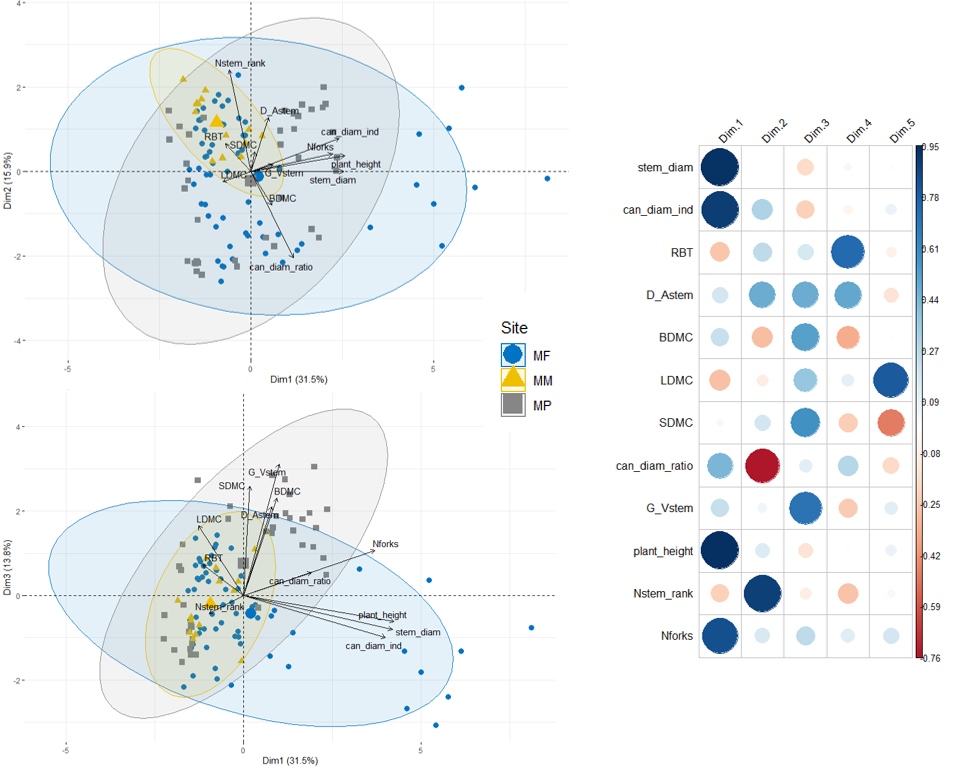


**Figure II.** Principal Component Analysis (PCA; upper biplot refers to dimensions 1-2, lower biplot to dimensions 1-3) ran on the twelve functional and architectural traits showing the occupancy (convex hull) of the three study sites in the multivariate space. On the right panel, trait loadings (correlation) on the five main dimensions of the PCA. MF = Montiferru; MM = Mt Morrone; MP = Mt Pisano.


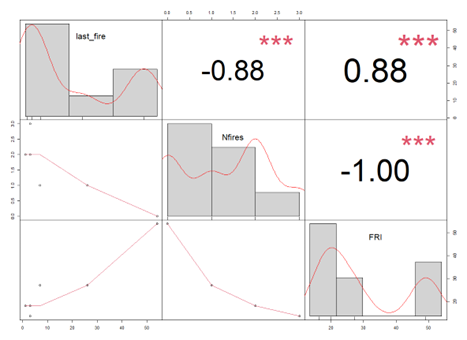


**Figure III.** Correlation (Sperman’s rho) between the three fire metrics used in this study: time since last fire event (last_fire), number of fires (Nfires), fire return interval (FRI).

**SUPPLEMENTARY MATERIAL 2**

SUMMARY STATISTICS OF MODELS DEVISED TO ADDRESS RESEARCH QUESTIONS 2-4.

FOR QUESTION 2

**Call: sma(formula = RBT + 1 ~ last_fire * species, data = data, log = "y",**

**method = c("OLS"), alpha = 0.05)**

Fit using Ordinary Least-Squares Regression

These variables were log-transformed before fitting: y

Confidence intervals (CI) are at 95%

------------------------------------------------------------

Cannot perform common slope test with method == "OLS" , use lm() instead.

Coefficients by group in variable "species"

Group: Cistus

elevation slope

estimate 0.5335216 -0.0031013006

lower limit 0.4539543 -0.0063442218

upper limit 0.6130889 0.0001416205

H0 : variables uncorrelated.

R-squared : 0.1205337

P-value : 0.060145

Group: Erica

elevation slope

estimate 0.5681179 -0.002442840

lower limit 0.5169207 -0.004147991

upper limit 0.6193150 -0.000737689

H0 : variables uncorrelated.

R-squared : 0.1812309

P-value : 0.0061679

Group: Quercus

elevation slope

estimate 0.5006778 -0.004340052

lower limit 0.3958235 -0.007393771

upper limit 0.6055321 -0.001286332

H0 : variables uncorrelated.

R-squared : 0.1453871

P-value : 0.0062945

**Call: sma(formula = D_Astem + 1 ~ last_fire * species, data = data,**

**log = "y", method = c("OLS"), alpha = 0.05)**

Fit using Ordinary Least-Squares Regression

These variables were log-transformed before fitting: y

Confidence intervals (CI) are at 95%

------------------------------------------------------------

Cannot perform common slope test with method == "OLS" , use lm() instead.

Coefficients by group in variable "species"

Group: Cistus

elevation slope

estimate 0.02121869 0.01927368

lower limit -0.19238348 0.01056791

upper limit 0.23482086 0.02797946

H0 : variables uncorrelated.

R-squared : 0.4234635

P-value : 9.8785e-05

Group: Erica

elevation slope

estimate 0.16457255 0.02509540

lower limit -0.06435104 0.01747096

upper limit 0.39349614 0.03271983

H0 : variables uncorrelated.

R-squared : 0.5388234

P-value : 7.0483e-08

Group: Quercus

elevation slope

estimate 0.7294441 -0.010370017

lower limit 0.4715358 -0.017881202

upper limit 0.9873525 -0.002858831

H0 : variables uncorrelated.

R-squared : 0.1383277

P-value : 0.0078249

**Call: sma(formula = BDMC ~ last_fire * species, data = data, log = "y",**

**method = c("OLS"), alpha = 0.05)**

Fit using Ordinary Least-Squares Regression

These variables were log-transformed before fitting: y

Confidence intervals (CI) are at 95%

------------------------------------------------------------

Cannot perform common slope test with method == "OLS" , use lm() instead.

Coefficients by group in variable "species"

Group: Cistus

elevation slope

estimate 2.733987 0.0010631389

lower limit 2.714440 0.0002664461

upper limit 2.753535 0.0018598316

H0 : variables uncorrelated.

R-squared : 0.2106427

P-value : 0.010738

Group: Erica

elevation slope

estimate 2.766533 -0.0001728584

lower limit 2.741724 -0.0009991355

upper limit 2.791342 0.0006534187

H0 : variables uncorrelated.

R-squared : 0.004697764

P-value : 0.67431

Group: Quercus

elevation slope

estimate 2.712166 0.0010490085

lower limit 2.681474 0.0001551708

upper limit 2.742857 0.0019428462

H0 : variables uncorrelated.

R-squared : 0.1039443

P-value : 0.022405

**Call: sma(formula = LDMC ~ last_fire * species, data = data, log = "y",**

**method = c("OLS"), alpha = 0.05)**

Fit using Ordinary Least-Squares Regression

These variables were log-transformed before fitting: y

Confidence intervals (CI) are at 95%

------------------------------------------------------------

Cannot perform common slope test with method == "OLS" , use lm() instead.

Coefficients by group in variable "species"

Group: Cistus

elevation slope

estimate 2.96971 -0.006789766

lower limit 2.92933 -0.008407919

upper limit 3.01009 -0.005171613

H0 : variables uncorrelated.

R-squared : 0.7329987

P-value : 3.1792e-09

Group: Erica

elevation slope

estimate 2.879916 1.183725e-03

lower limit 2.843594 -2.601321e-05

upper limit 2.916238 2.393462e-03

H0 : variables uncorrelated.

R-squared : 0.09359403

P-value : 0.054878

Group: Quercus

elevation slope

estimate 2.864443 -0.002007559

lower limit 2.844933 -0.002575770

upper limit 2.883954 -0.001439349

H0 : variables uncorrelated.

R-squared : 0.5125137

P-value : 5.0979e-09

**Call: sma(formula = SDMC ~ last_fire * species, data = data, log = "y",**

**method = c("OLS"), alpha = 0.05)**

Fit using Ordinary Least-Squares Regression

These variables were log-transformed before fitting: y

Confidence intervals (CI) are at 95%

------------------------------------------------------------

Cannot perform common slope test with method == "OLS" , use lm() instead.

Coefficients by group in variable "species"

Group: Cistus

elevation slope

estimate 2.689784 0.002468096

lower limit 2.661927 0.001332711

upper limit 2.717642 0.003603481

H0 : variables uncorrelated.

R-squared : 0.4145642

P-value : 0.00012354

Group: Erica

elevation slope

estimate 2.733051 1.011505e-03

lower limit 2.700667 -5.364525e-05

upper limit 2.765436 2.076655e-03

H0 : variables uncorrelated.

R-squared : 0.09096124

P-value : 0.062054

Group: Quercus

elevation slope

estimate 2.763692 -0.0006125483

lower limit 2.736922 -0.0013964010

upper limit 2.790463 0.0001713045

H0 : variables uncorrelated.

R-squared : 0.05104374

P-value : 0.12257

**Call: sma(formula = can_diam_ratio ~ last_fire * species, data = data,**

**log = "y", method = c("OLS"), alpha = 0.05)**

Fit using Ordinary Least-Squares Regression

These variables were log-transformed before fitting: y

Confidence intervals (CI) are at 95%

------------------------------------------------------------

Cannot perform common slope test with method == "OLS" , use lm() instead.

Coefficients by group in variable "species"

Group: Cistus

elevation slope

estimate -0.06900742 -0.002520184

lower limit -0.12701924 -0.004884569

upper limit -0.01099560 -0.000155799

H0 : variables uncorrelated.

R-squared : 0.1454862

P-value : 0.037548

Group: Erica

elevation slope

estimate -0.5143825 0.008891020

lower limit -0.5843183 0.006561767

upper limit -0.4444467 0.011220273

H0 : variables uncorrelated.

R-squared : 0.6111012

P-value : 2.6083e-09

Group: Quercus

elevation slope

estimate -0.3032328 0.005279606

lower limit -0.3565730 0.003726155

upper limit -0.2498926 0.006833057

H0 : variables uncorrelated.

R-squared : 0.4931117

P-value : 1.3242e-08

**Call: sma(formula = G_Vstem + 1 ~ last_fire * species, data = data,**

**log = "y", method = c("OLS"), alpha = 0.05)**

Fit using Ordinary Least-Squares Regression

These variables were log-transformed before fitting: y

Confidence intervals (CI) are at 95%

------------------------------------------------------------

Cannot perform common slope test with method == "OLS" , use lm() instead.

Coefficients by group in variable "species"

Group: Cistus

elevation slope

estimate 0.9722809 0.023328771

lower limit 0.5806805 0.007368329

upper limit 1.3638813 0.039289213

H0 : variables uncorrelated.

R-squared : 0.2425166

P-value : 0.0057003

Group: Erica

elevation slope

estimate 1.676128 0.007033357

lower limit 1.511812 0.001560712

upper limit 1.840444 0.012506002

H0 : variables uncorrelated.

R-squared : 0.1511973

P-value : 0.013149

Group: Quercus

elevation slope

estimate 0.1217432 0.011072005

lower limit -0.1423065 0.003381961

upper limit 0.3857929 0.018762048

H0 : variables uncorrelated.

R-squared : 0.1486393

P-value : 0.0056924

FOR QUESTION 3

*Erica*

**Call:**

**lm(formula = log10(plant_height) ~ log10(stem_diam) * last_fire,**

**data = dataE)**

Residuals:

Min 1Q Median 3Q Max

-0.266896 -0.027931 0.004746 0.061306 0.149846

Coefficients:

Estimate Std. Error t value Pr(>|t|)

(Intercept) 1.996063 0.022722 87.848 < 2e-16 ***

log10(stem_diam) 0.660082 0.065013 10.153 4.13e-12 ***

last_fire 0.007352 0.001853 3.968 0.000331 ***

log10(stem_diam):last_fire -0.008232 0.003026 -2.720 0.009981 **

---

Signif. codes: 0 ‘***’ 0.001 ‘**’ 0.01 ‘*’ 0.05 ‘.’ 0.1 ‘ ’ 1

Residual standard error: 0.1006 on 36 degrees of freedom

Multiple R-squared: 0.9116, Adjusted R-squared: 0.9042

F-statistic: 123.7 on 3 and 36 DF, p-value: < 2.2e-16

**Call:**

**lm(formula = log10(D_Astem + 1) ~ log10(RBT) * last_fire, data = dataE)**

Residuals:

Min 1Q Median 3Q Max

-0.91302 -0.28273 -0.04363 0.25753 0.86717

Coefficients:

Estimate Std. Error t value Pr(>|t|)

(Intercept) 0.67961 0.31299 2.171 0.0366 *

log10(RBT) -1.36561 0.74778 -1.826 0.0761 .

last_fire 0.01297 0.00709 1.829 0.0757 .

log10(RBT):last_fire 0.03843 0.01976 1.945 0.0596 .

---

Signif. codes: 0 ‘***’ 0.001 ‘**’ 0.01 ‘*’ 0.05 ‘.’ 0.1 ‘ ’ 1

Residual standard error: 0.4924 on 36 degrees of freedom

Multiple R-squared: 0.5851, Adjusted R-squared: 0.5505

F-statistic: 16.92 on 3 and 36 DF, p-value: 5.056e-07

**Call:**

**lm(formula = log10(G_Vstem + 1) ~ log10(plant_height) * last_fire,**

**data = dataE)**

Residuals:

Min 1Q Median 3Q Max

-1.3483 -0.0657 0.0320 0.1076 0.6968

Coefficients:

Estimate Std. Error t value Pr(>|t|)

(Intercept) -0.35888 0.52942 -0.678 0.50219

log10(plant_height) 1.01678 0.26221 3.878 0.00043 ***

last_fire 0.03402 0.03767 0.903 0.37244

log10(plant_height):last_fire -0.01530 0.01491 -1.026 0.31187

---

Signif. codes: 0 ‘***’ 0.001 ‘**’ 0.01 ‘*’ 0.05 ‘.’ 0.1 ‘ ’ 1

Residual standard error: 0.3129 on 36 degrees of freedom

Multiple R-squared: 0.4014, Adjusted R-squared: 0.3515

F-statistic: 8.046 on 3 and 36 DF, p-value: 0.0003131

**Call:**

**lm(formula = log10(can_diam_ratio + 1) ~ log10(SDMC) * last_fire,**

**data = dataE)**

Residuals:

Min 1Q Median 3Q Max

-0.08256 -0.02939 -0.01044 0.02683 0.09703

Coefficients:

Estimate Std. Error t value Pr(>|t|)

(Intercept) -1.019066 0.447005 -2.280 0.0288 *

log10(SDMC) 0.418767 0.163760 2.557 0.0150 *

last_fire 0.028675 0.012533 2.288 0.0283 *

log10(SDMC):last_fire -0.009509 0.004573 -2.080 0.0450 *

---

Signif. codes: 0 ‘***’ 0.001 ‘**’ 0.01 ‘*’ 0.05 ‘.’ 0.1 ‘ ’ 1

Residual standard error: 0.04579 on 35 degrees of freedom

(1 observation deleted due to missingness)

Multiple R-squared: 0.673, Adjusted R-squared: 0.645

F-statistic: 24.01 on 3 and 35 DF, p-value: 1.28e-08

*Quercus*

**Call:**

**lm(formula = log10(plant_height) ~ log10(stem_diam) * last_fire,**

**data = dataQ)**

Residuals:

Min 1Q Median 3Q Max

-0.43577 -0.08783 0.00133 0.10651 0.25745

Coefficients:

Estimate Std. Error t value Pr(>|t|)

(Intercept) 1.9357727 0.0353141 54.816 < 2e-16 ***

log10(stem_diam) 0.7402227 0.1005647 7.361 2.63e-09 ***

last_fire -0.0002032 0.0019240 -0.106 0.916

log10(stem_diam):last_fire -0.0002325 0.0025438 -0.091 0.928

---

Signif. codes: 0 ‘***’ 0.001 ‘**’ 0.01 ‘*’ 0.05 ‘.’ 0.1 ‘ ’ 1

Residual standard error: 0.1507 on 46 degrees of freedom

Multiple R-squared: 0.8465, Adjusted R-squared: 0.8364

F-statistic: 84.53 on 3 and 46 DF, p-value: < 2.2e-16

**Call:**

**lm(formula = log10(D_Astem + 1) ~ log10(RBT) * last_fire, data = dataQ)**

Residuals:

Min 1Q Median 3Q Max

-1.09286 -0.27446 -0.07209 0.16106 1.44014

Coefficients:

Estimate Std. Error t value Pr(>|t|)

(Intercept) 0.459890 0.106781 4.307 8.61e-05 ***

log10(RBT) 1.314341 0.212440 6.187 1.52e-07 ***

last_fire -0.006713 0.003126 -2.147 0.037080 *

log10(RBT):last_fire -0.028875 0.006938 -4.162 0.000137 ***

---

Signif. codes: 0 ‘***’ 0.001 ‘**’ 0.01 ‘*’ 0.05 ‘.’ 0.1 ‘ ’ 1

Residual standard error: 0.4895 on 46 degrees of freedom

Multiple R-squared: 0.5298, Adjusted R-squared: 0.4991

F-statistic: 17.28 on 3 and 46 DF, p-value: 1.183e-07

**Call:**

**lm(formula = log10(G_Vstem + 1) ~ log10(plant_height) * last_fire,**

**data = dataQ)**

Residuals:

Min 1Q Median 3Q Max

-1.39702 -0.29685 -0.14235 0.03521 1.40602

Coefficients:

Estimate Std. Error t value Pr(>|t|)

(Intercept) -0.58453 0.88318 -0.662 0.5114

log10(plant_height) 0.36787 0.42202 0.872 0.3879

last_fire -0.05586 0.02944 -1.897 0.0641 .

log10(plant_height):last_fire 0.02404 0.01215 1.979 0.0538 .

---

Signif. codes: 0 ‘***’ 0.001 ‘**’ 0.01 ‘*’ 0.05 ‘.’ 0.1 ‘ ’ 1

Residual standard error: 0.6042 on 46 degrees of freedom

Multiple R-squared: 0.3247, Adjusted R-squared: 0.2807

F-statistic: 7.373 on 3 and 46 DF, p-value: 0.0003908

**Call:**

**lm(formula = log10(can_diam_ratio + 1) ~ log10(SDMC) * last_fire,**

**data = dataQ)**

Residuals:

Min 1Q Median 3Q Max

-0.11078 -0.01921 0.01015 0.01028 0.11375

Coefficients:

Estimate Std. Error t value Pr(>|t|)

(Intercept) -1.406927 0.514217 -2.736 0.00893 **

log10(SDMC) 0.574885 0.186258 3.086 0.00350 **

last_fire 0.031486 0.011461 2.747 0.00868 **

log10(SDMC):last_fire -0.010663 0.004167 -2.559 0.01401 *

---

Signif. codes: 0 ‘***’ 0.001 ‘**’ 0.01 ‘*’ 0.05 ‘.’ 0.1 ‘ ’ 1

Residual standard error: 0.04311 on 44 degrees of freedom

(2 observations deleted due to missingness)

Multiple R-squared: 0.633, Adjusted R-squared: 0.6079

F-statistic: 25.29 on 3 and 44 DF, p-value: 1.149e-09

*Cistus*

**Call:**

**lm(formula = log10(plant_height) ~ log10(stem_diam) * last_fire,**

**data = dataC)**

Residuals:

Min 1Q Median 3Q Max

-0.30511 -0.07876 0.01418 0.09578 0.18654

Coefficients:

Estimate Std. Error t value Pr(>|t|)

(Intercept) 1.974640 0.066007 29.916 < 2e-16 ***

log10(stem_diam) 0.845938 0.177176 4.775 6.09e-05 ***

last_fire -0.002358 0.001657 -1.424 0.1665

log10(stem_diam):last_fire -0.010713 0.005026 -2.131 0.0427 *

---

Signif. codes: 0 ‘***’ 0.001 ‘**’ 0.01 ‘*’ 0.05 ‘.’ 0.1 ‘ ’ 1

Residual standard error: 0.1237 on 26 degrees of freedom

Multiple R-squared: 0.5764, Adjusted R-squared: 0.5275

F-statistic: 11.79 on 3 and 26 DF, p-value: 4.605e-05

**Call:**

**lm(formula = log10(D_Astem + 1) ~ log10(RBT + 1) * last_fire,**

**data = dataC)**

Residuals:

Min 1Q Median 3Q Max

-0.51986 -0.08879 -0.06141 -0.04925 1.63473

Coefficients:

Estimate Std. Error t value Pr(>|t|)

(Intercept) -0.059306 0.351034 -0.169 0.867

log10(RBT + 1) 0.160545 0.661284 0.243 0.810

last_fire 0.021495 0.013111 1.639 0.113

log10(RBT + 1):last_fire -0.005103 0.032565 -0.157 0.877

Residual standard error: 0.4746 on 26 degrees of freedom

Multiple R-squared: 0.4248, Adjusted R-squared: 0.3584

F-statistic: 6.4 on 3 and 26 DF, p-value: 0.002155

**Call:**

**lm(formula = log10(G_Vstem + 1) ~ log10(plant_height) * last_fire,**

**data = dataC)**

Residuals:

Min 1Q Median 3Q Max

-1.37487 -0.21240 0.02106 0.29338 0.95443

Coefficients:

Estimate Std. Error t value Pr(>|t|)

(Intercept) -6.36626 1.24640 -5.108 2.53e-05 ***

log10(plant_height) 4.34525 0.73388 5.921 3.02e-06 ***

last_fire 0.17891 0.06061 2.952 0.00661 **

log10(plant_height):last_fire -0.09239 0.03322 -2.781 0.00995 **

---

Signif. codes: 0 ‘***’ 0.001 ‘**’ 0.01 ‘*’ 0.05 ‘.’ 0.1 ‘ ’ 1

Residual standard error: 0.5684 on 26 degrees of freedom

Multiple R-squared: 0.6775, Adjusted R-squared: 0.6403

F-statistic: 18.21 on 3 and 26 DF, p-value: 1.429e-06

**Call:**

**lm(formula = log10(can_diam_ratio + 1) ~ log10(SDMC) * last_fire,**

**data = dataC)**

Residuals:

Min 1Q Median 3Q Max

-0.10684 -0.03880 0.01339 0.02871 0.06941

Coefficients:

Estimate Std. Error t value Pr(>|t|)

(Intercept) 0.952822 0.466711 2.042 0.0515 .

log10(SDMC) -0.253058 0.173050 -1.462 0.1556

last_fire -0.018352 0.059127 -0.310 0.7587

log10(SDMC):last_fire 0.006403 0.021182 0.302 0.7648

---

Signif. codes: 0 ‘***’ 0.001 ‘**’ 0.01 ‘*’ 0.05 ‘.’ 0.1 ‘ ’ 1

Residual standard error: 0.04969 on 26 degrees of freedom

Multiple R-squared: 0.2225, Adjusted R-squared: 0.1328

F-statistic: 2.481 on 3 and 26 DF, p-value: 0.08335

FOR QUESTION 4

*Erica*

**Call:**

**lm(formula = log10(plant_height) ~ last_fire * Nforks, data = dataE)**

Residuals:

Min 1Q Median 3Q Max

-0.45490 -0.05313 0.04006 0.12405 0.24899

Coefficients:

Estimate Std. Error t value Pr(>|t|)

(Intercept) 1.920473 0.041896 45.839 < 2e-16 ***

last_fire 0.011552 0.003307 3.493 0.001284 **

Nforks 0.123441 0.031686 3.896 0.000408 ***

last_fire:Nforks -0.002264 0.001061 -2.134 0.039751 *

---

Signif. codes: 0 ‘***’ 0.001 ‘**’ 0.01 ‘*’ 0.05 ‘.’ 0.1 ‘ ’ 1

Residual standard error: 0.1711 on 36 degrees of freedom

Multiple R-squared: 0.744, Adjusted R-squared: 0.7226

F-statistic: 34.87 on 3 and 36 DF, p-value: 9.516e-11

**Call:**

**lm(formula = log10(RBT) ~ last_fire * Nforks, data = dataE)**

Residuals:

Min 1Q Median 3Q Max

-0.42138 -0.11060 0.01372 0.09449 0.27588

Coefficients:

Estimate Std. Error t value Pr(>|t|)

(Intercept) 0.4185819 0.0407233 10.279 2.97e-12 ***

last_fire -0.0085452 0.0032147 -2.658 0.0116 *

Nforks 0.0429889 0.0307989 1.396 0.1713

last_fire:Nforks 0.0003633 0.0010312 0.352 0.7267

---

Signif. codes: 0 ‘***’ 0.001 ‘**’ 0.01 ‘*’ 0.05 ‘.’ 0.1 ‘ ’ 1

Residual standard error: 0.1663 on 36 degrees of freedom

Multiple R-squared: 0.2945, Adjusted R-squared: 0.2357

F-statistic: 5.009 on 3 and 36 DF, p-value: 0.005267

**Call:**

**lm(formula = log10(G_Vstem + 1) ~ last_fire * Nforks, data = dataE)**

Residuals:

Min 1Q Median 3Q Max

-1.64685 -0.03146 0.03110 0.20824 0.35747

Coefficients:

Estimate Std. Error t value Pr(>|t|)

(Intercept) 1.623865 0.087995 18.454 <2e-16 ***

last_fire 0.007662 0.006946 1.103 0.277

Nforks 0.109069 0.066550 1.639 0.110

last_fire:Nforks -0.002270 0.002228 -1.019 0.315

---

Signif. codes: 0 ‘***’ 0.001 ‘**’ 0.01 ‘*’ 0.05 ‘.’ 0.1 ‘ ’ 1

Residual standard error: 0.3594 on 36 degrees of freedom

Multiple R-squared: 0.2105, Adjusted R-squared: 0.1447

F-statistic: 3.199 on 3 and 36 DF, p-value: 0.03476

**Call:**

**lm(formula = log10(LDMC) ~ last_fire * Nforks, data = dataE)**

Residuals:

Min 1Q Median 3Q Max

-0.33901 -0.01935 0.00436 0.04211 0.08292

Coefficients:

Estimate Std. Error t value Pr(>|t|)

(Intercept) 2.8734325 0.0196995 145.863 <2e-16 ***

last_fire 0.0004916 0.0015551 0.316 0.754

Nforks 0.0170893 0.0148987 1.147 0.259

last_fire:Nforks -0.0001521 0.0004988 -0.305 0.762

---

Signif. codes: 0 ‘***’ 0.001 ‘**’ 0.01 ‘*’ 0.05 ‘.’ 0.1 ‘ ’ 1

Residual standard error: 0.08045 on 36 degrees of freedom

Multiple R-squared: 0.1353, Adjusted R-squared: 0.06319

F-statistic: 1.877 on 3 and 36 DF, p-value: 0.1509

*Quercus*

**Call:**

**lm(formula = log10(plant_height) ~ last_fire * Nforks, data = dataQ)**

Residuals:

Min 1Q Median 3Q Max

-0.60038 -0.12482 0.00881 0.16931 0.45682

Coefficients:

Estimate Std. Error t value Pr(>|t|)

(Intercept) 1.9865442 0.0664763 29.884 < 2e-16 ***

last_fire 0.0063448 0.0021222 2.990 0.00447 **

Nforks 0.0841282 0.0601219 1.399 0.16843

last_fire:Nforks 0.0004839 0.0012482 0.388 0.70003

---

Signif. codes: 0 ‘***’ 0.001 ‘**’ 0.01 ‘*’ 0.05 ‘.’ 0.1 ‘ ’ 1

Residual standard error: 0.2369 on 46 degrees of freedom

Multiple R-squared: 0.6208, Adjusted R-squared: 0.5961

F-statistic: 25.11 on 3 and 46 DF, p-value: 9.023e-10

**Call:**

**lm(formula = log10(RBT) ~ last_fire * Nforks, data = dataQ)**

Residuals:

Min 1Q Median 3Q Max

-0.88493 -0.28785 -0.03531 0.32569 0.86512

Coefficients:

Estimate Std. Error t value Pr(>|t|)

(Intercept) 0.233540 0.128763 1.814 0.0762 .

last_fire -0.010912 0.004111 -2.654 0.0109 *

Nforks 0.044686 0.116455 0.384 0.7030

last_fire:Nforks 0.000501 0.002418 0.207 0.8367

---

Signif. codes: 0 ‘***’ 0.001 ‘**’ 0.01 ‘*’ 0.05 ‘.’ 0.1 ‘ ’ 1

Residual standard error: 0.4588 on 46 degrees of freedom

Multiple R-squared: 0.198, Adjusted R-squared: 0.1457

F-statistic: 3.787 on 3 and 46 DF, p-value: 0.01648

**Call:**

**lm(formula = log10(G_Vstem + 1) ~ last_fire * Nforks, data = dataQ)**

Residuals:

Min 1Q Median 3Q Max

-1.3404 -0.2306 -0.1444 -0.1144 1.5838

Coefficients:

Estimate Std. Error t value Pr(>|t|)

(Intercept) 0.1142057 0.1706733 0.669 0.507

last_fire 0.0001767 0.0054487 0.032 0.974

Nforks 0.0470028 0.1543590 0.305 0.762

last_fire:Nforks 0.0036357 0.0032046 1.135 0.262

Residual standard error: 0.6081 on 46 degrees of freedom

Multiple R-squared: 0.3158, Adjusted R-squared: 0.2712

F-statistic: 7.079 on 3 and 46 DF, p-value: 0.0005212

**Call:**

**lm(formula = log10(LDMC) ~ last_fire * Nforks, data = dataQ)**

Residuals:

Min 1Q Median 3Q Max

-0.090171 -0.035155 0.004995 0.038528 0.089855

Coefficients:

Estimate Std. Error t value Pr(>|t|)

(Intercept) 2.866e+00 1.393e-02 205.687 < 2e-16 ***

last_fire -1.776e-03 4.448e-04 -3.993 0.000233 ***

Nforks -2.224e-03 1.260e-02 -0.177 0.860669

last_fire:Nforks -6.146e-05 2.616e-04 -0.235 0.815275

---

Signif. codes: 0 ‘***’ 0.001 ‘**’ 0.01 ‘*’ 0.05 ‘.’ 0.1 ‘ ’ 1

Residual standard error: 0.04964 on 46 degrees of freedom

Multiple R-squared: 0.5219, Adjusted R-squared: 0.4907

F-statistic: 16.74 on 3 and 46 DF, p-value: 1.72e-07

*Cistus*

**Call:**

**lm(formula = log10(plant_height) ~ last_fire * Nstem_rank, data = dataC)**

Residuals:

Min 1Q Median 3Q Max

-0.40669 -0.09050 0.01800 0.09672 0.26002

Coefficients:

Estimate Std. Error t value Pr(>|t|)

(Intercept) 1.522417 0.084274 18.065 3.09e-16 ***

last_fire 0.004555 0.004057 1.123 0.2718

Nstem_rank 0.111759 0.051633 2.164 0.0398 *

last_fire:Nstem_rank -0.001589 0.001333 -1.192 0.2441

---

Signif. codes: 0 ‘***’ 0.001 ‘**’ 0.01 ‘*’ 0.05 ‘.’ 0.1 ‘ ’ 1

Residual standard error: 0.1592 on 26 degrees of freedom

Multiple R-squared: 0.298, Adjusted R-squared: 0.217

F-statistic: 3.679 on 3 and 26 DF, p-value: 0.02476

**Call:**

**lm(formula = log10(RBT + 1) ~ last_fire * Nstem_rank, data = dataC)**

Residuals:

Min 1Q Median 3Q Max

-0.49095 -0.09950 0.01664 0.11439 0.25235

Coefficients:

Estimate Std. Error t value Pr(>|t|)

(Intercept) 0.4087841 0.0896803 4.558 0.000108 ***

last_fire -0.0004266 0.0043173 -0.099 0.922045

Nstem_rank 0.0843043 0.0549449 1.534 0.137025

last_fire:Nstem_rank -0.0017144 0.0014185 -1.209 0.237711

---

Signif. codes: 0 ‘***’ 0.001 ‘**’ 0.01 ‘*’ 0.05 ‘.’ 0.1 ‘ ’ 1

Residual standard error: 0.1694 on 26 degrees of freedom

Multiple R-squared: 0.1942, Adjusted R-squared: 0.1012

F-statistic: 2.088 on 3 and 26 DF, p-value: 0.1262

**Call:**

**lm(formula = log10(G_Vstem + 1) ~ last_fire * Nstem_rank, data = dataC)**

Residuals:

Min 1Q Median 3Q Max

-1.33006 -0.64645 -0.06419 0.55993 1.27754

Coefficients:

Estimate Std. Error t value Pr(>|t|)

(Intercept) -0.094686 0.395393 -0.239 0.81262

last_fire 0.057529 0.019035 3.022 0.00557 **

Nstem_rank 0.700973 0.242247 2.894 0.00761 **

last_fire:Nstem_rank -0.017364 0.006254 -2.776 0.01005 *

---

Signif. codes: 0 ‘***’ 0.001 ‘**’ 0.01 ‘*’ 0.05 ‘.’ 0.1 ‘ ’ 1

Residual standard error: 0.747 on 26 degrees of freedom

Multiple R-squared: 0.443, Adjusted R-squared: 0.3787

F-statistic: 6.893 on 3 and 26 DF, p-value: 0.001443

**Call:**

**lm(formula = log10(LDMC) ~ last_fire * Nstem_rank, data = dataC)**

Residuals:

Min 1Q Median 3Q Max

-0.196823 -0.024155 0.003694 0.014749 0.125308

Coefficients:

Estimate Std. Error t value Pr(>|t|)

(Intercept) 2.914e+00 3.636e-02 80.128 < 2e-16 ***

last_fire -2.757e-05 1.717e-03 -0.016 0.98731

Nstem_rank 2.717e-02 2.191e-02 1.240 0.22648

last_fire:Nstem_rank -2.079e-03 5.645e-04 -3.683 0.00111 **

---

Signif. codes: 0 ‘***’ 0.001 ‘**’ 0.01 ‘*’ 0.05 ‘.’ 0.1 ‘ ’ 1

Residual standard error: 0.06678 on 25 degrees of freedom

(1 observation deleted due to missingness)

Multiple R-squared: 0.8447, Adjusted R-squared: 0.8261

F-statistic: 45.33 on 3 and 25 DF, p-value: 2.948e-10
